# Improved remediation of co-contaminated soils by heavy metals and PAHs with biosurfactant-enhanced soil washing

**DOI:** 10.1038/s41598-022-07577-7

**Published:** 2022-03-08

**Authors:** Xu Zhang, Xiaodong Zhang, Shuguang Wang, Shan Zhao

**Affiliations:** School of Environmental Science and Engineering, Shandong University, Qingdao, 266237 People’s Republic of China

**Keywords:** Biomaterials, Environmental biotechnology

## Abstract

Due to the huge toxicity of co-contaminated soil with PAHs and heavy metals and the complexity of their remediation, it is thus critical to take effective remediation actions to remove heavy metals and PAHs simultaneously from the co-contaminated soil. Biosurfactant-enhanced soil washing (BESW) were investigated in this study for remediation of soil co-contaminated with phenanthrene (PHE) and cadmium (Cd). The co-existence of PHE and Cd caused the change of the structure of soil and rhamnolipid micelle, which lead to different removal rate of PHE and Cd from co-contaminated soil compared with single contaminated soil. The results of FT-IR and NMR showed that PHE entered micelles of rhamnolipid and Cd formed the complexation with the external carboxyl groups of rhamnolipid micelle. We also found that pH, concentration of rhamnolipid solution, temperature and ionic strength had influence on co-contaminated soil remediation. The effects of above mentioned four factors on co-contaminated soil remediation in BESW processes were analyzed by using Taguchi design of experiment method. Taguchi based Grey Relational Analysis was conducted to identify the optimal remediation conditions, which included pH = 9, concentration of rhamnolipid = 5 g/L, temperature = 15 °C and ionic strength = 0.01 M. Under the optimal conditions for BESW, removal rates of cadmium and phenanthrene reached 72.4% and 87.8%, respectively in co-contaminated soil.

## Introduction

Soil acts as a final acceptor of substances that are released into the environment from various human activities^1^. Soil contamination has received more and more attentions globally, especially at industrial sites such as petrochemical refinery and former manufactured gas plant sites^2,3^. There are a variety of contaminants in soil at these sites instead of single contaminant, where co-existence of heavy metals and polycycle aromatic hydrocarbons (PAHs) are prevalent^4–6^. Co-contamination by heavy metals and PAHs in soil has posed more serious threats to eco-environment and human health, such as increased toxicity to microbial activity and diversity, lowered bioavailability and inhibited plant growth^7–10^. Coexistence of PAHs and heavy metals may affect their and soil’s physical and chemical properties, consequently making co-contaminated soil remediation become more difficult^11–14^. It is thus critical to take effective remediation actions to remove heavy metals and PAHs simultaneously from the co-contaminated soil.

Previously, a number of remediation methods and techniques have been developed for remediating the soil contaminated by co-contaminants, such as electrokinetic remediation, soil washing, phytoremediation and bioremediation^15–18^. Among them, soil washing is regarded as one of the highly efficient and cost-effective techniques due to its flexibility in remediation of a wide range of pollutants^19–21^. It can be used to remove heavy metals and PAHs in soil through the mechanisms of mobilization and solubilization. In order to improve the remediation performance, various washing agents have been developed and used in soil washing, such as humic acids^22,23^, vegetable oils^24,25^, chelating agents^26,27^, water-miscible co-solvents^28^, and surfactants^29–33^.

Although chelating agents such as [S,S]-ethylene diamine disuccinate (EDDS) and Ethylene Diamine Tetraacetic Acid (EDTA) were effective for removal of heavy metals, they encountered difficulties in removing organic compounds^34^. In order to remediate PAHs and heavy metals co-contaminated soil, synthetic surfactants were frequently combined with chelating agents^35–39^. However, these synthetic surfactants as extracting agents could have irreversible effects on physio-chemical properties of soil such as loss of soil organic matter and essential nutrients, and at the same time, most of them were ineffective in removing heavy metals due to their weak electrostatic attraction and coordination between surfactants and heavy metals^21,40^. As a result, selection of effective and biodegradable agents in soil washing is crucial in simultaneous remediation of the soil co-contaminated by heavy metals and PAHs.

As alternatives to synthetic surfactants, biosurfactants have been widely used as enhancing agents for removing heavy metals or PAHs from soil due to their advantages in higher biodegradability, foamability and selectivity for heavy metals and organic compounds, lower toxicity, better environmental compatibility^41–47^. Using biosurfactants as washing agents in soil washing has become a viable option in simultaneously removing heavy metals and PAHs from co-contaminated soil, leading to biosurfactant enhanced soil washing (BESW).

Therefore, the objective of this study is to investigate the performance of biosurfactant enhanced soil washing in remediation of soil co-contaminated by PAHs and heavy metals. Removal mechanisms of BESW for simultaneous remediation of PAHs and heavy metals as well as the effects of key factors on BESW processes are analyzed. Optimal remediation conditions of BESW are identified using Taguchi design of experiment method. The results of this study will provide insight into remediation of co-contaminated soil caused by heavy metals and PAHs.

## Materials and methods

### Chemical and materials

Phenanthrene and Cd(NO_3_)_2_, nitric acid, sulfuric acid, methanol, methylene chloride, ethanol, rhamnolipid, sodium chloride and sodium hydroxide were purchased from Sinopharm Chemical Reagent Co., Ltd. (Shanghai, China). All reagents were of analytical purity grade. Deionized water used in this study was obtained from a Millipore Milli-Q system.

### Soil preparation

The clean sand was collected from the beach close to the east gate of Shandong University, Qingdao, China. The clean Kaolinite was purchased from Shanghai Alighting Biochemical Technology Co., Ltd. Sand and Kaolinite were mixed with a mass ratio of 2:1 to form the uncontaminated soil, which was air-dried and sieved through a 100-mesh sieve. Samples of co-contaminated soil were prepared by dissolving phenanthrene and cadmium in methyl alcohol (500 mL) and then mixing this solution with soil. These mixtures were then shaken evenly and placed in a shaker at 150 rpm, 25 °C for 24 h; samples of co-contaminated soil were finally air-dried in a fume hood for a week. The concentration of PHE and Cd after spiking and aging showed < 5% deviation from the targeted spiked concentration. To investigate the effect of pollutants co-existence on soil, we compared the removal rates of pollutants in co-contaminated and individual-contaminated soils. Six types of contaminated soils were prepared in this study since the concentrations of co-contaminants in soil might affect the removal of cadmium or phenanthrene. These soil samples were designated SC0P2, SC2P0, SC1P2, SC2P2, SC2P1, where C and P denotes the concentration of Cd and PHE in soil (0 represents 0 mg/kg; 1 represents 40 mg/kg; 2 represents 200 mg/kg), respectively.

### Batch experiment

Biosurfactants are amphiphilic compounds which comprise two major components including hydrophobic (i.e. water insoluble tail group) and hydrophilic (i.e. water soluble head group). Biosurfactants are useful to promote the mobilization of hydrophobic compounds absorbed onto soil particles by reducing surface and interfacial tension. Rhamnolipid as a representative biosurfactant has many advantages such as low toxicity, high biodegradability, ecological safety, low CMC and possibility to be produced in contaminated sites^48^. It exhibited a higher extraction efficiency than synthetic surfactants in the removal of petroleum hydrocarbons from soils^43^. Rhamnolipid has more functional groups and lager molecular structures, which can effectively solubilize and desorb heavy metals^49^. Thus, rhamnolipid is used in biosurfactant enhanced soil washing. Four sets of experiments were conducted to examine the effects of four influencing factors on rhamnolipid enhanced removal of PHE and Cd. The four factors include initial pH and ion strength of rhamnolipid solution, concentration of rhamnolipid, and temperature. The individual effects of these four factors on removal of PHE and Cd were firstly tested. The 2 g of co-contaminated soil and 20 mL of rhamnolipid solution were mixed in the borosilicate glass vials and then shaken at 200 rpm for 24 h at a temperature of 25 °C. The initial pH values of solutions were adjusted to be in a range of 3 to 11 by titration with H_2_SO_4_ and NaOH, which were measured using a BPH-20 Meter. To investigate the effects of inorganic salt ions, different concentrations of NaCl were separately added into rhamnolipid solution.

### Analytical methods

All the above-mentioned tests were performed in triplicate. After being shaken, samples were put into a centrifuge at 4000 rpm for 20 min to separate the supernatant. Phenanthrene were extracted from supernatant by n-hexane, which used liquid–liquid extraction. All extracts were dried by rotary evaporation and then re-dissolved in equal volume of methanol. These samples were then analyzed using HPLC with a mobile phase of methanol–water (v/v, 80/20) at a flow rate of 1 ml min^−1^. The wavelength of the detector was set to be 251 nm. At the same time, we added 5 mL of HNO_3_ into supernatant. Then organic matter in supernatant was removed by heating. The concentrations of Cd were analyzed by atomic absorption analysis (AAS). The phenanthrene or cadmium removal percentages were calculated by the following equations:

1 $${\mathrm{R}}_{\mathrm{phe}}\left(\mathrm{\%}\right)=\frac{{\mathrm{C}}_{\mathrm{phe}}\mathrm{V}}{{\mathrm{m}}_{\mathrm{phe}}}*100\mathrm{\%}$$

2 $${\mathrm{R}}_{\mathrm{cd}}\left(\mathrm{\%}\right)=\frac{{\mathrm{C}}_{\mathrm{cd}}\mathrm{V}}{{\mathrm{m}}_{\mathrm{cd}}}*100\mathrm{\%}$$

where R_phe_ and R_cd_ are the removal efficiency of phenanthrene and cadmium from soil (%), respectively; C_phe_ and C_cd_ are the concentration of phenanthrene and cadmium in the eluent (mg/L), respectively; V is the volume of the eluent (L); m_phe_ and m_cd_ are total amount of phenanthrene and cadmium in soil (mg), respectively.

### ^1^H nuclear magnetic resonance (NMR) experiments

The supernatant after soil washing was mixed with D_2_O, of which 600 μl were moved to NMR tubes. NMR analyses conducted on the pure rhamnolipid solution were used to compare the hydrogen bond changes of rhamnolipid after elution. Proton NMR chemical shift measurements were carried out at 400 MHz on a Bruker spectrometer. The peak of D_2_O was 4.81 ppm in all spectra.

### Taguchi experimental design

Taguchi design of experiments (DOE) approach is effective to optimize process parameters^50^. In various disciplines, the Taguchi DOE method was used to address the impacts of process parameters on the mean and identify the significant variables^51^. The factors or parameters with different levels are predetermined based on the single factor experiments. Using the Taguchi method, an experimental design consisting of an orthogonal array can be produced. In this study, four most important factors including ion strength, pH and concentration of rhamnolipid solution, and temperature were used as input variables for Taguchi DOE, where their levels were determined based on trial experiments. A design of L9(3^4^) orthogonal arrays was employed in this study, where L represented the orthogonal design including nine experimental tests with three levels for each of the four factors. Table 1 shows the detailed design of experiments. The signal-to-noise (S/N) ratio was introduced to analyze the effects of each factor’s level on the removal rate in Taguchi DOE. Generally, there are three methods to calculate S/N ratios including LB (Lower is Better), NB (Nominal is better) and HB (Higher or larger is Better)^52,53^. Since removal rates should always be maximized, the HB approach for S/N ratio was used as follows:

**Table 1:** The orthogonal experimental design (L_9_(3^4^)).

Testing number	Factor A	Factor B	Factor C	Factor D
	pH	Concentration	Temperature	Ion strength
1	A1 (3)	B1 (1 g/L)	C1 (15 °C)	D1 (0.01 M)
2	A1 (3)	B2 (3 g/L)	C2 (25 °C)	D2 (0.03 M)
3	A1 (3)	B3 (5 g/L)	C3 (35 °C)	D3 (0.05 M)
4	A2 (7)	B1 (1 g/L)	C2 (25 °C)	D3 (0.05 M)
5	A2 (7)	B2 (3 g/L)	C3 (35 °C)	D1 (0.01 M)
6	A2 (7)	B3 (5 g/L)	C1 (15 °C)	D2 (0.03 M)
7	A3 (9)	B1 (1 g/L)	C3 (35 °C)	D2 (0.03 M)
8	A3 (9)	B2 (3 g/L)	C1 (15 °C)	D3 (0.05 M)
9	A3 (9)	B3 (5 g/L)	C2 (25 °C)	D1 (0.01 M)

3 $$\frac{S}{N}=-10log10(\frac{1}{n}\sum \frac{1}{{yi}^{2}})$$

where *n* is the number of repetitions under the same experimental conditions; and *y* represents the measured results for removal efficiency.

### Grey relational analysis

Grey relational analysis (GRA) is used to evaluate the degree of relevance between influencing factors and behavior. Firstly, the analysis results of the responses need to be normalized in the range of [0, 1]. In the Taguchi-based grey relational analysis, the grey relational coefficient (ζi) is used to determine the optimal parameters and can be classified into three categories: the-nominal-the-better, the-lower-the-better and the-higher-the better^54^. In this work, “large is better” is used for both objective functions because it is desired to maximize PHE and Cd removal rates. The original sequence is normalized as follows^55^:

4 $${y}_{i}\left(k\right)= \frac{{x}_{i}^{0}\left(k\right)-min {x}_{i}^{0}(k)}{max{x}_{i}^{0}\left(k\right)-min{x}_{i}^{0}(k)}$$

where ${y}_{i}(k)$ represents the normalization value of grey relational generation; $max {x}_{i}^{0}(k)$ and $min{x}_{i}^{0}(k)$ mean the maximum and minimum value of ${x}_{i}^{0}(k)$, respectively; and ${x}^{0}$ denotes the optimum value.

After the normalization process, the grey relational coefficient (ζ_i_) needs to be calculated using Eqs. (5)–(8) to address the relationship between the ideal and actual normalized value^56^:

5 $${\zeta }_{i}\left(k\right)=\frac{{\Delta }_{min}+\psi {\Delta }_{max}}{{\Delta }_{0i}\left(k\right)+\psi {\Delta }_{max}}$$

6 $${\Delta }_{0i}=\left|\left|{y}_{0}\left(k\right)-{y}_{i}\left(k\right)\right|\right|$$

7 $${\Delta }_{max}= \begin{array}{cc}max& max\\ \forall j\in i& \forall k\end{array}\left|\left|{y}_{0}\left(k\right)-{y}_{i}\left(k\right)\right|\right|$$

8 $${\Delta }_{min}=\begin{array}{cc}min& min\\ \forall j\in i& \forall k\end{array}\left|\left|{y}_{0}\left(k\right)-{y}_{i}\left(k\right)\right|\right|$$

where ψ is the identification coefficient that is limited in the range 0 < ψ < 1. In the GRA, taking any identification coefficient value between 0 and 1 does not change the order of importance of the parameters. In general, ψ = 0.5 is used^57,58^. ${\Delta }_{0i}$ is the deviation value between ${y}_{0}(k)$ and ${y}_{i}(k)$. ${y}_{0}\left(k\right)$ is the referential sequence and ${y}_{i}(k)$ is the comparative sequence. ${\Delta }_{max}$ and ${\Delta }_{min}$ is the maximum and minimum value of the ${\Delta }_{0i}$, respectively^55^.

The degree of grey relational grade (GRG) is calculated as follows^59^:

9 $${\gamma }_{i}\left(k\right)=\frac{1}{m}\sum \limits_{k=1}^{m}{\lambda }_{k}{\gamma }_{i}(k)$$

where m is the number of samples and n is the number of related factor sequences. In the BEWS process for remediation of co-contaminated soil, since the removal rates of PHE and Cd are equally important, the weight ratios of PHE and Cd removal rates are same (i.e., ${\lambda }_{k}=0.5$).

### Adsorption isotherm

The adsorption isotherms were investigated under different concentrations of phenanthrene and cadmium. Batch experiments were conducted to determine phenanthrene and cadmium equilibrium sorption to soil. In each of 50-mL brown glass bottles, 2 g of clean soil sample was dissolved into 20 mL of Cd(NO_3_)_2_ solution or phenanthrene solution with different concentrations. These mixtures were shaken at 200 rpm for a period of 24 h at 25 °C After equilibrium, the tubes were centrifuged at 4000 rpm for 20 min to separate solid and aqueous phases. Supernatant was withdrawn for HPLC analysis of equilibrium phenanthrene concentration and for AAS analysis of equilibrium cadmium concentration. These mixtures were shaken at 200 rpm for a period of 24 h at 25 °C. The samples were then put into a centrifuge at 4000 rpm for 20 min to obtain supernatant. All the experiments were conducted in triplicate. The Freundlich and Langmuir models were used to address the adsorption characteristics of cadmium and phenanthrene by soil, which were expressed as follows:

The Freundlich model:

10 $${Q}_{e}={K}_{f}{C}_{e}n$$

The Langmuir model:

11 $${Q}_{e}=\frac{{Q}_{m}{k}_{L}{C}_{e}}{1+{k}_{L}{C}_{e}}$$

where *Q*_*e*_ is the absorbed amounts of pollutants by soil (mg kg^−1^); *C*_*e*_ is the equilibrium concentration (mg L^−1^); *K*_*f*_ is the Freundlich affinity coefficient; n is the exponential coefficient indicating isotherm nonlinearity; *k*_*L*_ is the Langmuir affinity coefficient; and *Q*_*m*_ is the maximum absorbed amounts of pollutants to soil (mg kg^−1^).

### Spectroscopic analysis

Fluorescence spectra of phenanthrene and its inclusion complex in aqueous solutions with different concentrations of rhamnolipid were recorded with a Hitachi F4600 fluorescence spectrophotometer; the fluorescence intensity was measured in a 1 cm quartz cell. Excitation wavelength used was set at 249 nm. The UV absorbance spectra of rhamnolipid solution, Cd (NO_3_)_2_ solution and rhamnolipid-cadmium solution were recorded with UV spectrophotometer (UV 8000, Shanghai Metash Instruments, China).

#### Characterization of rhamnolipid solution

The particle hydrodynamic diameter was determined using dynamic light scattering (DLS) and the zeta potential was determined using laser Doppler electrophoresis in a ZetaSizer Nano ZS (Malvern, UK).

## Results analyses

### Simultaneous removal of phenanthrene and cadmium

**Figure 1:**
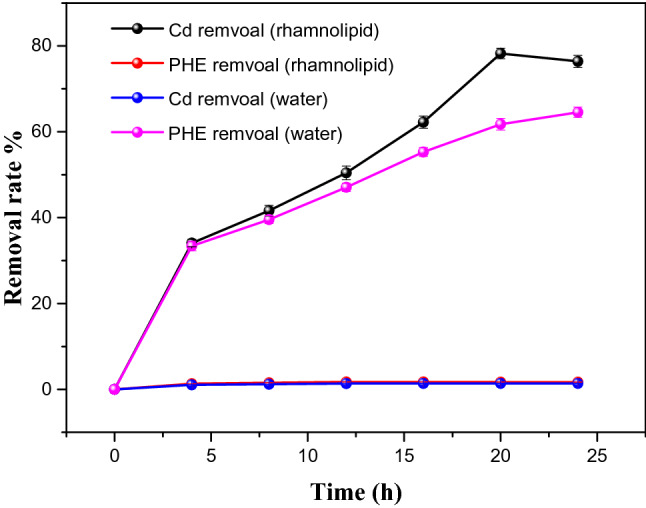
Removal rate of phenanthrene and cadmium from co-contaminated soil in different systems.

**Figure 2:**
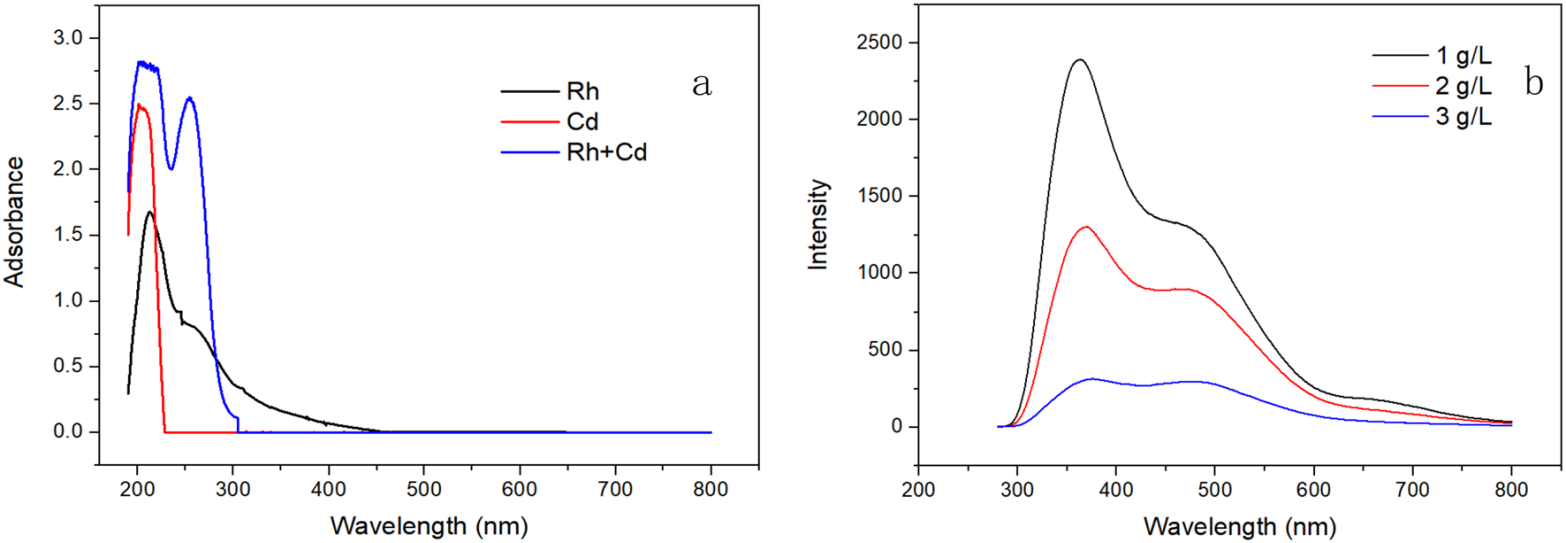
(**a**) Absorbance spectra of Rhamnolipid, Cadmium (Cd), and rhamnolipid-cadmium mixture (Rh + Cd) (^47^ = 0.3 g/L; [Cd] = 0.05 g/L); (**b**) The fluorescence spectra of phenanthrene/rhamnolipid inclusion complex in different concentrations of rhamnolipid ([PHE] = 10 mg/L).

Figure 1 shows the removal rates of phenanthrene and cadmium from co-contaminated soil in different systems. Deionized water had basically no effects on the removal of pollutants. When rhamnolipid was added, the removal rates of cadmium and phenanthrene increased by 76.5% and 64.55%, respectively. Those indicated that the presence of rhamnolipid can simultaneously promote the mobilization of phenanthrene and cadmium absorbed on soil. Rhamnolipid formed inclusion complex with phenanthrene to improved their solubility in aqueous solution. The inclusion complex was characterized with fluorescence spectra as shown in Fig. 2b. With the increase of rhamnolipid’s concentration, the fluorescence intensity of PHE decreased. It proved that a higher concentration of rhamnolipid led to formation of more micelles for providing more hydrophobic sites. It means more phenanthrene can entered the aqueous solution. The UV spectrum comparison results showed that the absorption peak appeared at approximately 275 nm in the rhamnolipid-cadmium mixed solution. As shown in Fig. 2a, the spectral changes indicated the formation of rhamnolipid-cadmium complex. Rhamnolipid reduced interfacial tension to promote complexes desorbed from soil. Cadmium removal in the process of rhamnolipid-enhanced soil washing may involve two mechanisms. Firstly, functional groups of rhamnolipid binds to cadmium through chelation and electrostatic adsorption. Secondly, rhamnolipid reduces interfacial tension and transforms cadmium into aqueous organic phases through formation of micelles or complexes*.*

**Figure 3:**
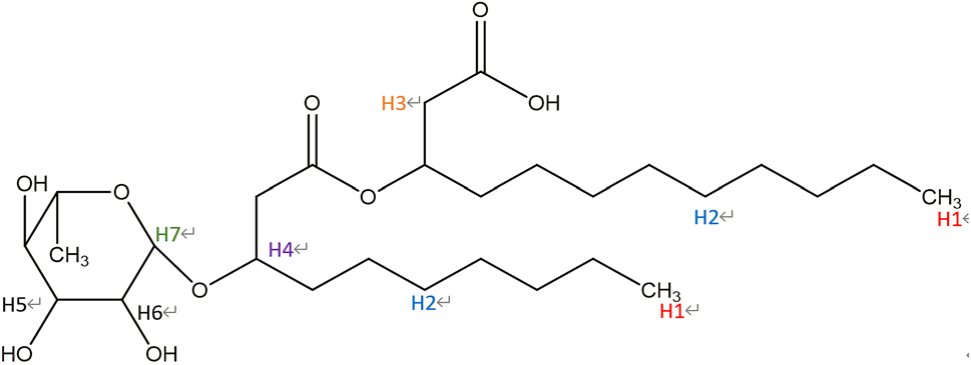
The structure of rhamnolipid.

**Figure 4:**
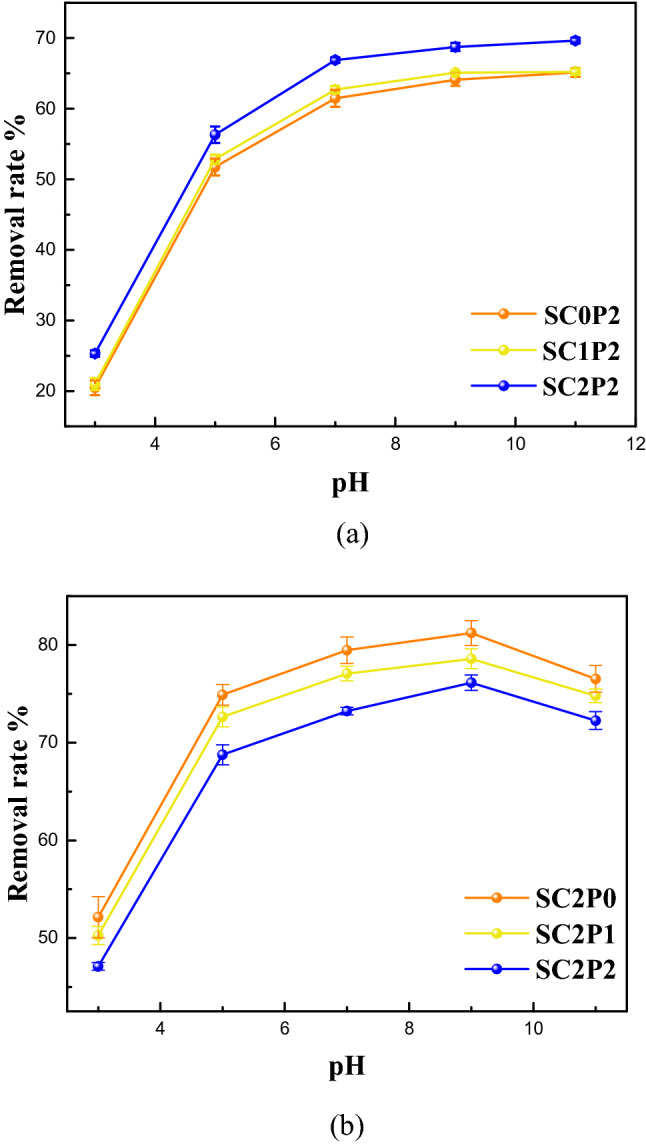
Effects of co-existence of pollutants on removal rate of cadmium or phenanthrene (C: cadmium added; P: phenanthrene added; 0: 0 mg/kg; 1: 40 mg/kg; 2: 200 mg/kg). (**a**) Removal rate of PHE in different soil. (**b**) Removal rate of Cd in different soil.

The different results of remediation of PHE-Cd co-contaminated soil or individual polluted soil are shown in Fig. 4. They showed the effects of co-existence of cadmium and phenanthrene on their removal efficiencies. Co-existing contaminants may compete the dissolution sites of the surfactant micelle, which may decrease pollutants’ solubility within micelles^60^. Co-existence of multiple contaminants can decrease the micelle-water interfacial tension, leading to loosened micelles and more room for solubilization of contaminants^60,61^. Therefore, the solubilization sites of pollutants within micelles would affect the desorption of pollutions significantly. The properties of the contaminant could determine sites at which the contaminant bound to rhamnolipid. Figure 3 shows the structure of rhamnolipid. By using NMR analyses, we obtained the proton chemical shift values of pure rhamnolipid and used rhamnolipid in soil washing (Table 2). When the rhamnolipid solution was mixed with phenanthrene or/and cadmium, different pollutants reacted with rhamnolipid at different locations; the presence of proton chemical shifts indicated the amounts of solute near that segment^62^. The capacity of soil to absorb pollution also affects the removal rates of pollutants. Therefore, Tables 3 and 4 showed the models and parameters related to adsorption isotherm of cadmium and phenanthrene in different soil samples.

**Table 2:** Proton chemical shifts (ppm) for rhamnolipid before and after soil washing.

Segment	Pure	PHE	Cd
H1	0.747	0.736	0.742
H2	1.155	1.147	1.151
H3	2.325	2.317	2.313
H4	3.336	3.332	3.330
H5	3.582	3.575	3.571
H6	3.941	3.936	3.998
H7	5.165	5.159	5.155

**Table 3:** Models and parameters related to adsorption isotherm of phenanthrene.

Model	Langmuir model			Freundlich model		
Parameter	*Q*_*m*_	*k*_*L*_	R^2^	*1/n*	*K*_*f*_	R^2^
Clean soil	140.85	0.046	0.928	0.572	2.96	0.980
Cd-contaminated soil	144.93	0.051	0.921	0.555	3.12	0.981

**Table 4:** Models and parameters related to adsorption isotherm of cadmium.

Model	Langmuir model			Freundlich model		
Parameter	*Q*_*m*_	*k*_*L*_	R^2^	*1/n*	*K*_*f*_	R^2^
Clean soil	454.55	0.367	0.998	0.133	11.33	0.986
PHE-contaminated soil	526.32	0.404	0.999	0.149	11.85	0.944

**Figure 5:**
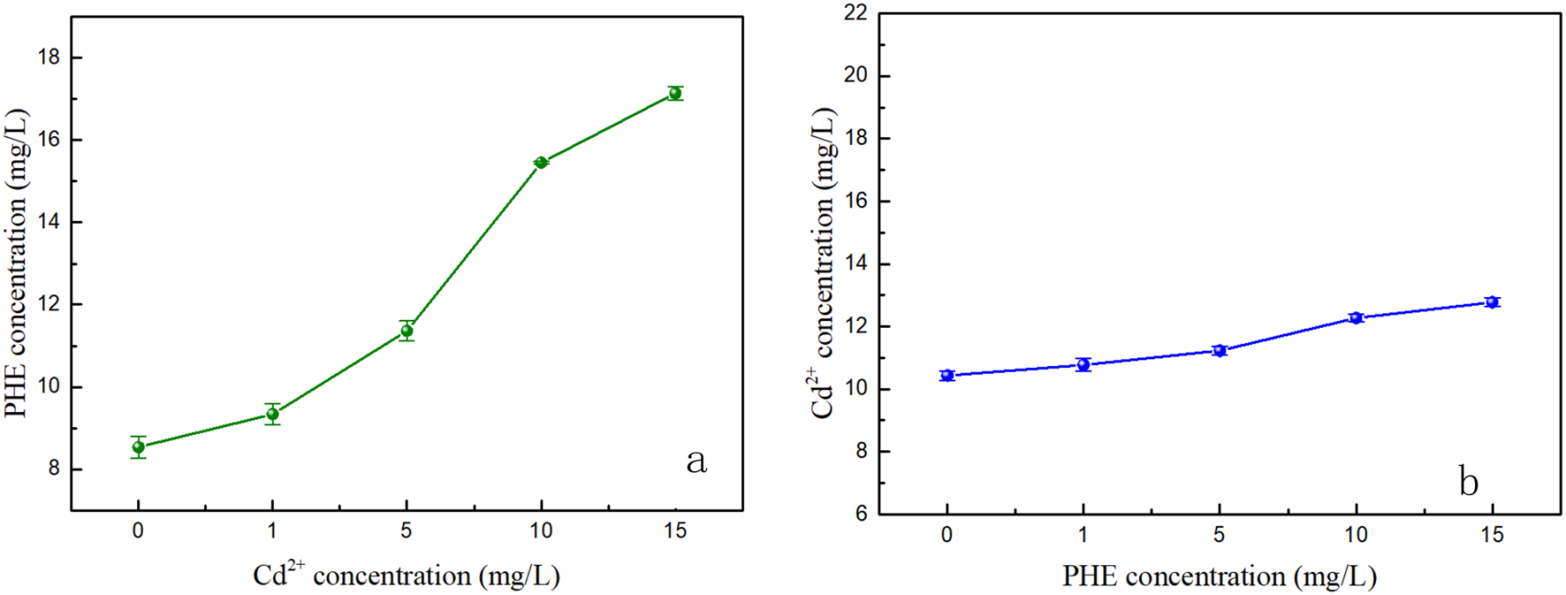
(**a**) Solubilization capacities of phenanthrene after rhamnolipid chelating with cadmium. (**b**) Chelation capacity for cadmium after the solubilization of phenanthrene.

Figure 4a showed the higher the concentration of cadmium in soil, the higher the removal rates of phenanthrene from co-contaminated soil. As shown in Table 2, we compared the 1H NMR spectroscopy of the pure rhamnolipid solution and rhamnolipid solution combined with cadmium or phenanthrene. The 1H chemical shift values for rhamnolipid demonstrated that phenanthrene was mainly solubilized in the micelle core due to the appearance of upward chemical shifts of H_1_ pertaining to hydrophobic chain. A large chemical shift in the hydrogen at a certain position of rhamnolipid molecule indicated a large amount of solutes nearby. At the same time, there were upward shifts in the protons on the hydrophobic chain (i.e., H_2_, H_3_), representing the existence of phenanthrene in both the micelle core and the palisade layer. In the presence of cadmium, upward chemical shifts of H_5_ and H_6_ pertaining to hydrophilic chain indicated complexation of some cadmium with hydroxyl. In the 1H NMR spectroscopy of rhamnolipid solution with cadmium, the adsorption peak of hydrogen on the hydroxyl group basically disappeared, further indicating the complexation of cadmium and hydroxyl. Meanwhile, the slight upward shifts of the protons on the hydrophobic chain (H_3_) showed that only a small amount of Cd^2+^ was complexed with the carboxyl group. It indicated that the combination sites of Cd and PHE were different. The adsorption isotherm of phenanthrene shown in Table 3 indicated that the Freundlich model well fitted the isotherms, with an adjusted coefficient of determination (R^2^) being larger than 0.98. The maximum adsorption capacity of phenanthrene to the soil with the presence of Cd^2+^ was larger than that to clean soil. On the one hand, the presence of heavy metals led to more energy sites in soil, thus increasing the adsorption capacity of PAHs^63,64^; on the other hand, formation of cation-Π bonding by heavy metals and PAHs could enhance the sorption affinities of PAHs in soil^65^. Figure 5a indicated cadmium entering rhamnolipid solution increased the solubility of phenanthrene in rhamnolipid solution. Cadmium reduced the electrostatic repulsion between the rhamnolipid molecules enabling the core volume to increase to some extent, consequently leading to dissolution of more phenanthrene. More micelles in solution which can provide more hydrophobic sites improved the desorption of phenanthrene from soil. Thus, the combined effect of adsorption capacity and solubility lead to the higher cadmium removal in co-contaminated soil.

Figure 4b showed a reduced tendency of cadmium removal as the concentration of phenanthrene increased. Table 5 shows the parameters of absorption isotherm of cadmium. The values of the correlation coefficient (R^2^) of the Freundlich model were lower than those of the Langmuir model, indicating Langmuir model was more suitable to describe the adsorption process of cadmium. Soil with phenanthrene displayed much higher adsorption capacity than those of clean soil under the same conditions. Cadmium and phenanthrene in soil might form complex compounds, making the desorption of cadmium more difficult. The results of 1H NMR spectroscopy analyses indicated large amounts of cadmium in the rhamnolipid fat head and most phenanthrene in the rhamnolipid hydrophobic tail. We observed that there was no competition between phenanthrene and cadmium, representing that phenanthrene may not affect the removal rate of cadmium in rhamnolipid enhanced soil washing. But Fig. 5b showed the formed complex after the solubilization of phenanthrene by rhamnolipid could slightly increase the chelation amount of cadmium, which may be due to the cation-Π interactions. Therefore, the increased adsorption capacity played a dominant role in the change of cadmium removal.

**Table 5:** ANOVA for phenanthrene and cadmium removal.

Pollution	Source	DF	SS	F-value	p	Contribution %
Phenanthrene	pH	2	1289.176	1050.959	0	0.8102
	Concentration	2	245.476	200.117	0	0.1544
	Temperature	2	23.479	19.140	0	0.0148
	Ion strength	2	20.519	16.727	0	0.0129
	Error	18	11.040			0.0069
Cadmium	pH	2	4284.147	3168.157	0	0.9345
	Concentration	2	230.57	170.486	0	0.0500
	Temperature	2	47.033	34.781	0	0.0100
	Ion strength	2	7.801	5.769	0.012	0.0014
	Error	18	12.170			0.0027

F_0.05_ (2,18) = 3.55.

### Effects of operational parameters on soil washing

Many factors may influence the performance of biosurfactant-enhanced soil washing, including temperature, pH, ion strength and concentration of rhamnolipid. In co-contaminated soil, the effects of the abovementioned parameters on the removal efficiency of pollutants will vary. To determine optimal operational conditions for BESW, we explored the influence of these factors on the removal rates of heavy metals and PAHs.

#### Effect of initial pH of the rhamnolipid solution

**Figure 6:**
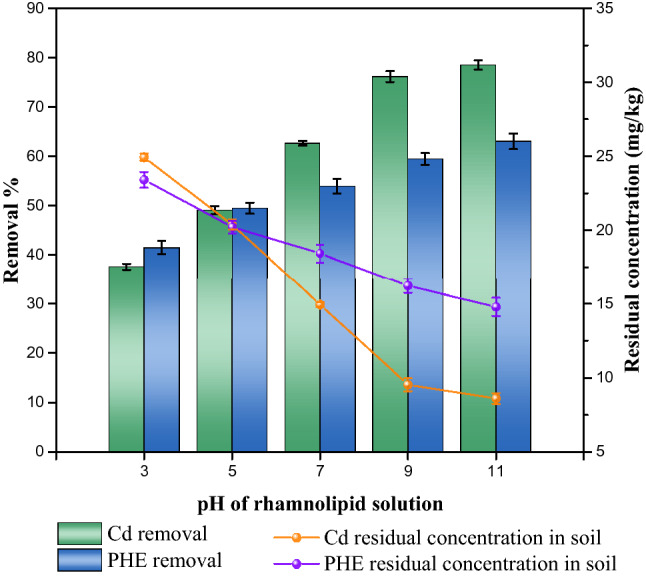
Effect of initial pH on removal of cadmium and phenanthrene in biosurfactant-enhanced soil washing process.

**Figure 7:**
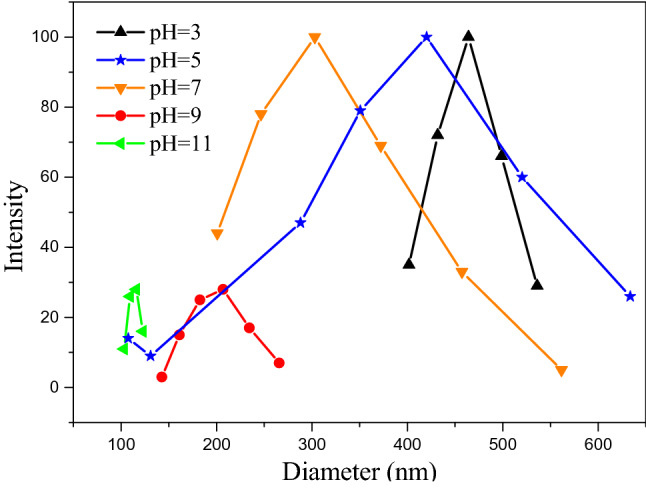
Effect of pH on the diameter of rhamnolipid.

Figure 6 shows the variation trends of cadmium and phenanthrene removal rates with the change of the initial pH value of the rhamnolipid solution. The phenanthrene’s removal efficiency increased as the pH value of the rhamnolipid solution increased due to the change of the morphology of rhamnolipid micelles. Rhamnolipid precipitated with a pH value of less than 5. When the pH value of the solution was greater than 5, the micelle structure of rhamnolipid changed from thin sheets to vesicles and finally into micelles, indicating the gradually decreased size of its structure^66^. Those were consistent with the sizes of micelle diameters at different pH values as shown in Fig. 7. As the pH value increased, rhamnolipid formed more micelles, providing more hydrophobic points for phenanthrene. The initial pH of the rhamnolipid solution also affected the soil absorption of rhamnolipid. Previous studies indicated the addition of NaOH could reduce the adsorption of surfactin in soil^67^. As a result, more rhamnolipids were used to remove the pollutants as the initial pH of the solution increased.

**Figure 8:**
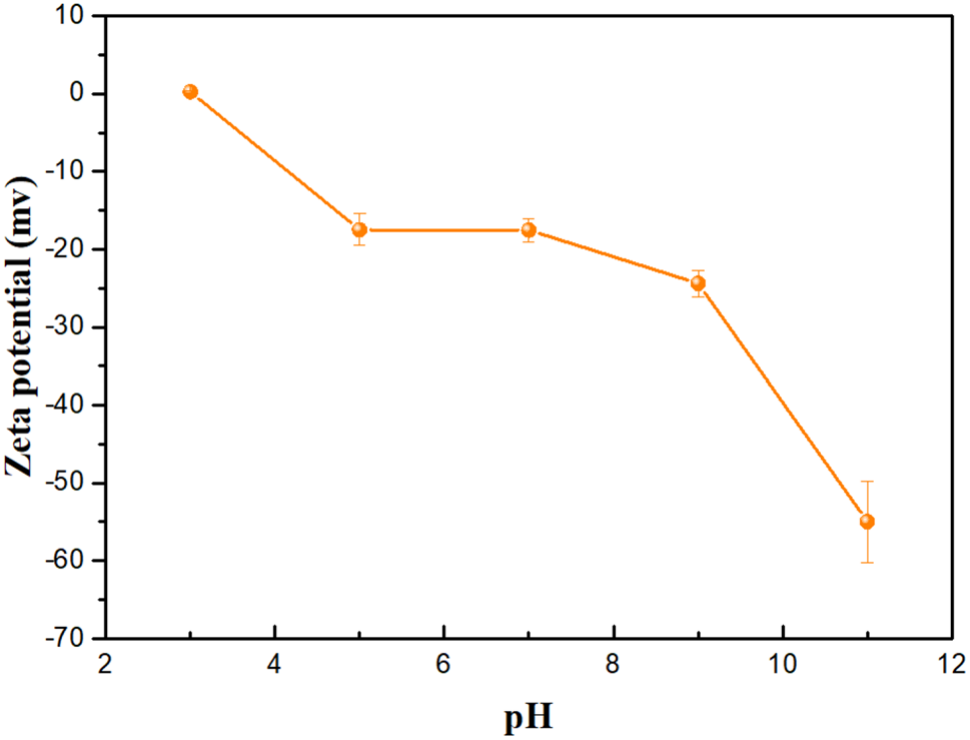
Effect of pH on the zeta potential of rhamnolipid solution.

The removal rate of cadmium increased as the pH value increased from 3 to 9, and slightly decreased when the pH value increased from 9 to 11. This indicated that strong acidic and alkaline conditions could weaken the ability of rhamnolipid to wash cadmium. Figure 8 showed that changes in the rhamnolipid solution pH from 3 to 11 had effects on the electrical potential of rhamnolipid, ranging from 0.2305 (pH = 3) to − 54.9667 (pH = 11). It indicated the electrostatic field strength around the rhamnolipid molecule decreased in the lower pH. Suspensions with zeta potential values lower than ± 20 mV are considered unstable and indicate a low rhamnolipid aggregation capacity due to the equilibrium of the charges^68^. An increase of the pH value of the rhamnolipid solution could promote the ionization of the carboxy and hydroxy portion in the solution. Washing of cadmium by rhamnolipid in soil depended on the complexation between cadmium ions and carboxyl groups or hydroxyl groups. An increase of the pH value led to more micelle and carboxyl groups and hydroxyl groups, consequently forming more complexation with cadmium. However, strong alkaline conditions could inhibit the desorption of heavy metals and reduce the chelation ability of rhamnolipid^69^. As a result, rhamnolipid-enhanced soil washing could well remediate cadmium and phenanthrene co-contaminated soil under neutral and weak-alkaline conditions.

#### Effect of temperature

**Figure 9:**
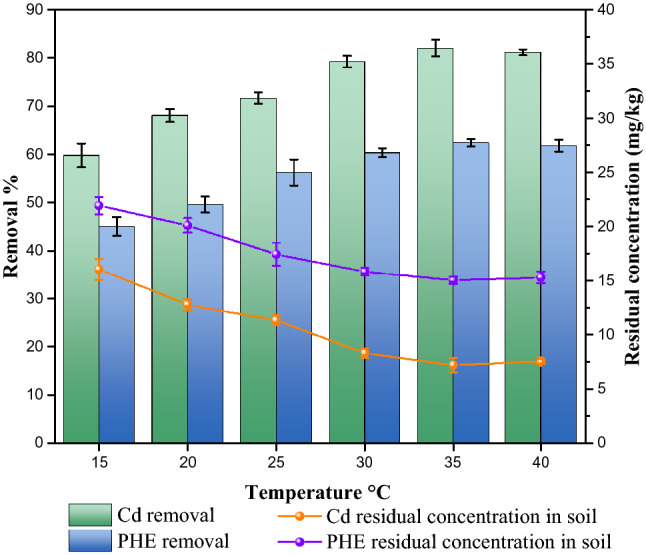
Effect of temperature on removal of cadmium and phenanthrene in biosurfactant-enhanced soil washing process.

Figure 9 presents the relationships between temperature and the removal rates of cadmium and phenanthrene. As the temperature increased from 15 to 40 °C, both the removal rates of cadmium and phenanthrene firstly increased sharply and then decreased slightly. Those demonstrated that the temperature had a significant impact on the desorption of cadmium and phenanthrene in the presence of rhamnolipid^70^. An increased temperature could lead to decreased hydration of the hydrophilic group and consequently higher solubility of PAHs and heavy metals. In addition, increasing temperature resulted in constantly accelerated movement of molecules and increased contact opportunities between rhamnolipid and cadmium or phenanthrene in soil so that it was easier for cadmium and phenanthrene to enter the aqueous phase. However, as the temperature increased from 35 to 40 °C, the removal rates of phenanthrene and cadmium slightly decreased. Wu et al.^71^ showed temperature affected the aggregation behavior of rhamnolipid. The increase of temperature is conductive to the transformation of rhamnolipid micelles into vesicles. Compared with micelles, vesicles are not conductive to the desorption of phenanthrene from soil and the decrease of surface tension. Therefore, the removal of phenanthrene and cadmium decreased at 40 °C.

#### Effect of the concentration of the rhamnolipid solution

**Figure 10:**
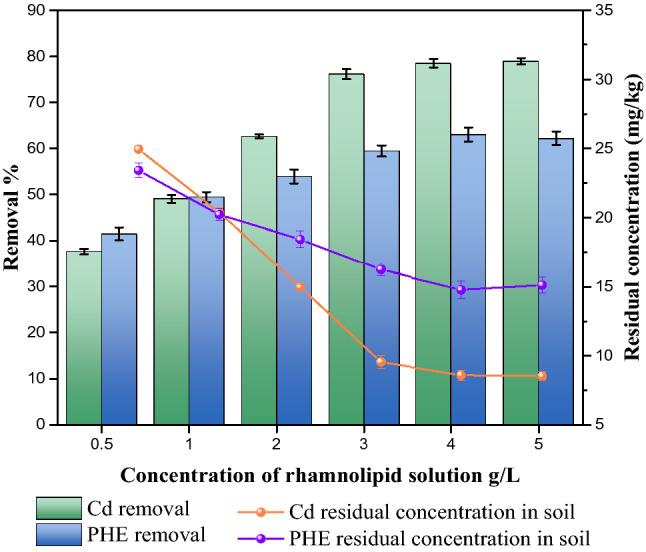
Effect of concentration of rhamnolipid on removal of cadmium and phenanthrene in biosurfactant-enhanced soil washing process.

The effect of the concentration of the rhamnolipid on the removal rates of cadmium and phenanthrene is shown in Fig. 10. The concentration of the rhamnolipid solution is an important factor in biosurfactant-enhanced soil washing. Previous studies indicated surfactants could reduce the interfacial tension between liquid and heavy metals to enhance the mobility of heavy metals^72^. As the concentration of the rhamnolipid solution increased from 0.5 to 4 g/L, the washing rate of cadmium increased from 37.5 to 78.7%. With the increase of the concentration of the rhamnolipid solution, more rhamnolipid molecules were introduced in the surfactant-enhanced soil washing process, leading to increased removal rate of cadmium. That’s due to the facts that (a) the carboxyl bound to cadmium ions through chelation; (b) the rhamnolipid molecular in the lipid and soil interface promoted the desorption of cadmium ions; and (c) rhamnolipid dispersed the soil by electrostatic repulsion. Similarly, an increase of the concentration of the rhamnolipid solution also led to the increased removal rate of phenanthrene due to the enhanced desorption of phenanthrene. As the concentration of the rhamnolipid solution increased from 0.5 to 0.4 g/L, removal rate of phenanthrene increased from 41.5 to 63.2%. Our results were consistent with the reported solubilization capacity of rhamnolipid^73^. However, when the concentration of the rhamnolipid solution exceeded 0.4 g/L, the removal rate of phenanthrene slightly decreased since more rhamnolipid molecules were absorbed by soil, leading to enhanced hydrophobicity of soil. It’s thus essential to choose an appropriate concentration of the rhamnolipid solution in order to improve the removal rates of phenanthrene and cadmium in the co-contaminated soil.

#### Effect of ionic strength of rhamnolipid solution

**Figure 11:**
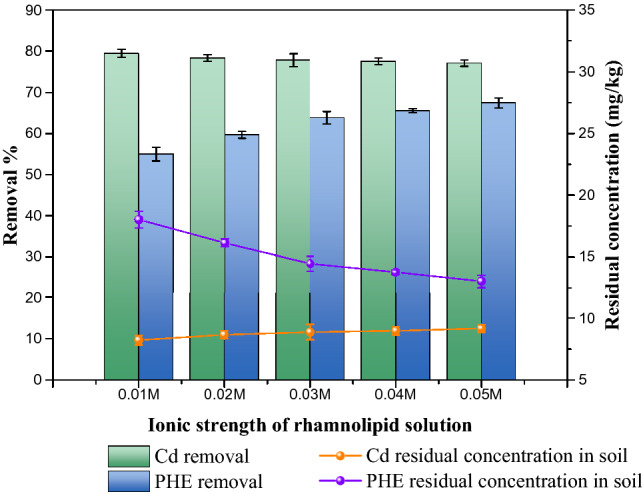
Effect of ionic strength of rhamnolipid solution on removal of cadmium and phenanthrene in biosurfactant-enhanced soil washing process.

Figure 11 shows the effects of variations of ionic strength of the rhamnolipid solution on removal rates of phenanthrene and cadmium. When the ionic strength of the rhamnolipid solution increased from 0.01 to 0.05 M, removal rate of phenanthrene increased from 63.1 to 66%. We added electrolytes to the solution of rhamnolipid, which led to the screening effect on the electrostatic repulsion between the charged aggregates. That promoted the formation of micelle and the solubility of rhamnolipid^74^. The binding of NaCl with carboxyl groups resulted in the formation of solvated groups^75^, indicating rhamnolipid desorbed from soil easily and more lipid rhamnolipid entered in the aqueous solution. As a result, increasing the ionic strength of the rhamnolipid solution had a positive effect on removal of phenanthrene. The effect of ionic strength of the rhamnolipid solution on cadmium’s removal was not significant, although the removal rate of cadmium decreased when rhamnolipid solution contained lots of NaCl. That’s since too much sodium competed with cadmium for binding sites on rhamnolipid.

### Optimization of the operation conditions in BESW

**Figure 12:**
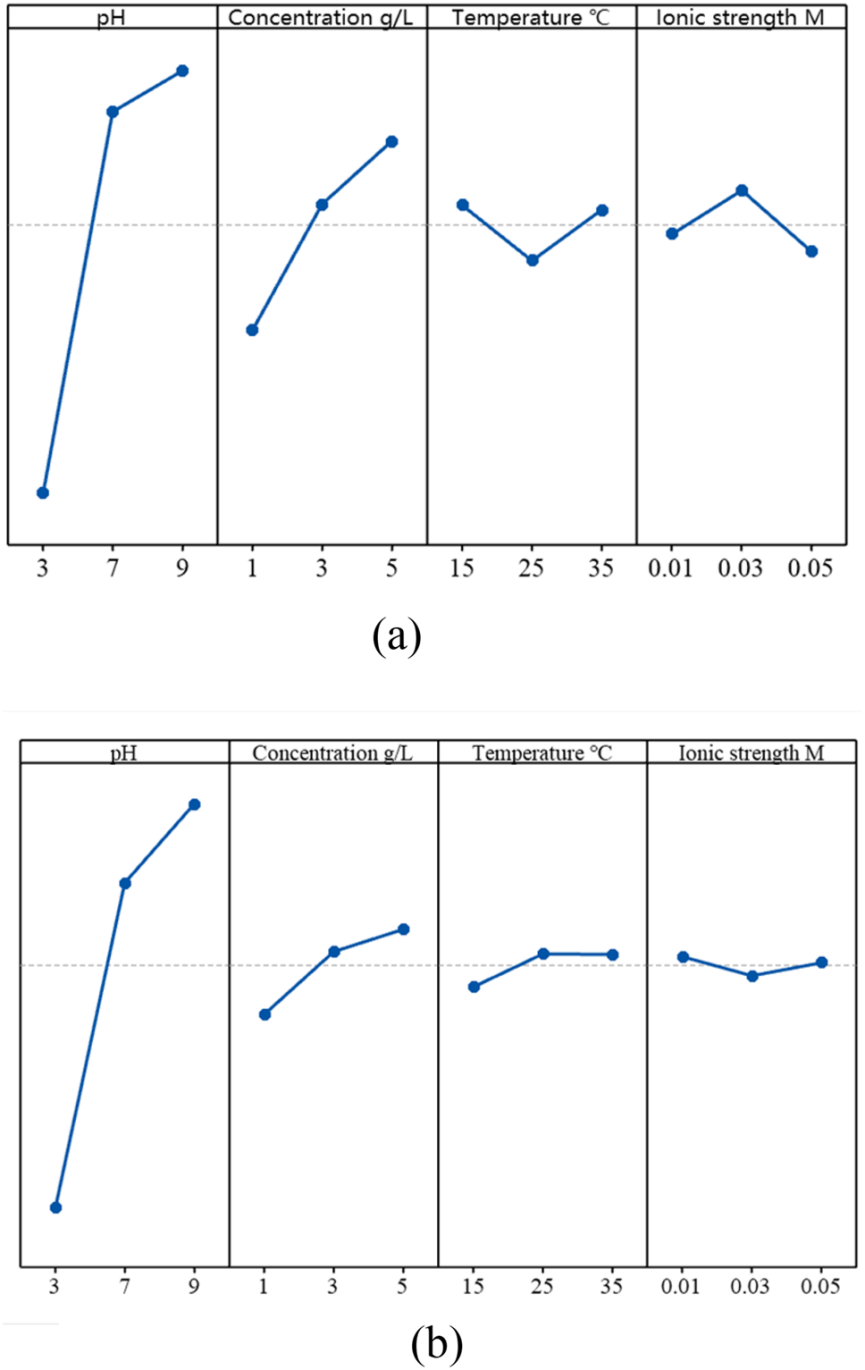
Main effect plots for signal-to-noise ratios for PHE and Cd removal rates. (**a**) S/N ratios for PHE removal rate. (**b**) S/N ratios for Cd removal rate.

**Figure 13:**
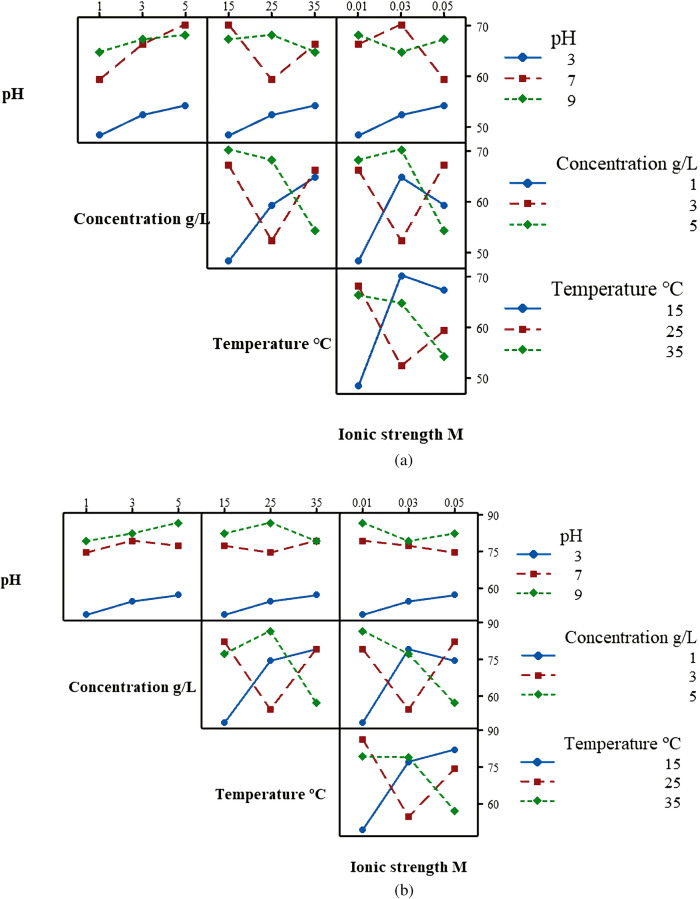
Interaction plot for removal rate vs pH, concentration of rhamnolipid, temperature and ionic concentration. (**a**) Interaction plot for removal rate of phenanthrene. (**b**) Interaction plot for removal rate of cadmium.

Figure 12a shows S/N ratios of different conditions for removal rates of phenanthrene. The S/N ratio represents each parameter’s impact on the PHE removal rate. The optimum conditions of the system are obtained from the level of factors which have the maximum S/N ratio. Comparison of single factor experiments with Taguchi DOE experiments indicated there had different change trends of PHE removal. For example, in the single factor experiment, with the increase of temperature from 15 to 35 °C, the removal rate of PHE increased. However, in the Taguchi DOE experiments when temperature was 15 °C, the S/N ratio for PHE removal rate was the highest. The optimum values of parameters for maximum removal rate of PHE (i.e., 64.55%) are determined as pH = 9, concentration = 0.5 g/L, temperature = 35 °C, and ionic strength = 0.05 M in the single factor experiments. However, analyses of S/N ratio of PHE removal rate indicated different optimal conditions such as pH = 9, concentration = 0.5 g/L, temperature = 15 °C, and ionic strength = 0.03 M; under these conditions, the removal rate of PHE was 72.2% (shown in Table 6). As a result, interactions of different factors on co-contamination remediation performance need to be considered in optimization of the BESW process. Figure 12a showed the effects of the interactions of multiple factors on removal rate of PHE. The interaction graphs indicated the slight interactions between pH and other three factors, while there were strong interactions between concentration of rhamnolipid, temperature and ionic concentration of rhamnolipid solution. For example, with the increase of ionic strength of rhamnolipid solution, the tendencies of PHE removal rate were different under different temperature due to the interaction between temperature and ionic strength. When the temperature was 15 °C and the ionic strength was 0.03 M, the removal rate of PHE was maximized. With the interaction plots for removal of PHE (Fig. 13a), interactions of the factors and the optimal conditions for the PHE removal could be obtained. In order to determine the relative significance and the contribution of individual factors on the removal rate of phenanthrene and cadmium, analysis of variance^76^ was carried out using SPSS. The critical value at α level of 0.05 could be found from the distribution table of the F-value, which was 3.55. A factor is considered significant if its F-value is greater than 3.55. From Table 5, we could find the F values of all factors exceeded the critical value of 3.55, indicating that pH, concentration of rhamnolipid solution, and ionic strength could significantly affect the removal of PHE. Furthermore, the percentage of contribution in the Table 5 showed the degree of effect of each factor on the process of BESW. The contribution percentage of pH, concentration of rhamnolipid solution, temperature and ionic strength of rhamnolipid solution in PHE removal rate was found to be 81.02%, 15.44%, 1.48% and 1.29%, respectively. The pH had the greatest impact on the removal rate of PHE, followed by concentration of rhamnolipid solution, temperature and ionic strength of rhamnolipid. These results agree with the result of the S/N ratios.

**Table 6:** Results of Taguchi DOE.

Testing number	PHE removal rate %	Cd removal rate %
1	48.2	49.1
2	52.3	54.6
3	54.2	57.1
4	59.3	74.4
5	66.3	79.3
6	70.3	77.2
7	64.8	79.1
8	67.3	82.2
9	68.2	86.6

Figure 12b suggests that Cd removal rate increases with an increase of pH from 5 to 9. The increased concentration of rhamnolipid solution and temperature could lead to increased removal rate of Cd. The removal rate of Cd decreased slightly as ionic strength of rhamnolipid solution increased from 0.01 to 0.03 M but increased at 0.05 M. Optimum parameters for removal rate of Cd were determined based on the highest S/N ratio for all factors and levels, which were pH = 9, concentration of rhamnolipid solution 0.5%, temperature = 25 °C, and ionic strength of rhamnolipid solution = 0.01 M; under these conditions, 86.6% of Cd was removed from co-contaminated soil (Table 6). Figure 13b showed the interaction graph of each factor. In the process of BESW, the interactions among concentration of rhamnolipid, temperature and ionic strength of rhamnolipid solution had a great influence on the performance of co-contamination remediation. However, there were few crossings in the interaction graph between pH and other factors. It indicated weak interactions between pH and other factors in the process of BESW. Based on ANOVA analysis, all four factors had significant impacts on the removal rate of Cd. The contribution of evaluated factors toward the removal rate of Cd is also shown in Table 5. Results showed that pH was the most significant factor exerting an influence on removal rate of Cd (93.45%), followed by concentration of rhamnolipid solution (5%), temperature (1%) and ionic strength of rhamnolipid solution (0.14%).

Since the optimum remediation conditions based on the S/N ratio were different for PHE and Cd removal under different situations, multi-objective optimization was employed for obtaining the optimum conditions for the whole BESW system by using GRA. Table 7 showed the gray correlation coefficient (ζ) and gray correlation degree ($\gamma )$ of PHE and Cd removal rates. The correlation of the comprehensive target decreased with the decrease of the correlation degree. The mean of GRG for all the experiments was obtained and shown in Table 8. The higher the mean of GRG, the better the co-contaminated soil remediation performance. The optimum BESW conditions for removing PHE and Cd were pH = 9, rhamnolipid solution concentration = 5 g/L, temperature = 15 °C, and ionic strength of rhamnolipid solution = 0.01 M; under these conditions, removal rates of PHE and Cd reached 72.4% and 84.8%, respectively.

**Table 7:** Relationship between gray correlation coefficient and gray correlation degree.

	ζ_PHE_	ζ_Cd_	$\gamma$(GRG)
1	0.33	0.33	0.33
2	0.38	0.37	0.37
3	0.40	0.39	0.40
4	0.50	0.61	0.55
5	0.73	0.72	0.73
6	1	0.67	0.83
7	0.67	0.71	0.69
8	0.79	0.81	0.80
9	0.84	1	0.92

**Table 8:** Main effects on Grey Relational Grades.

	A (pH)	B (concentration)	C (temperature)	D (ionic strength)
Level 1	0.369	0.526	**0.655**	**0.660**
Level 2	0.705	0.633	0.616	0.633
Level 3	**0.803**	**0.717**	0.605	0.583
Delta	0.434	0.191	0.05	0.077
Optimum	3	5 g/L	15 °C	0.01 M

Optimum remediation conditions in different parameters are presented in bold.

## Conclusions

Biosurfactant enhanced soil washing is an efficient and cost-effective technology for remediation of co-contaminated soil by PAHs and heavy metals. The main mechanisms of BESW for remediating co-contaminated soil by PHE and Cd is investigated in this study, where rhamnolipid increases the solubility of PHE and Cd complexes with rhamnolipid. Co-existence of PHE and Cd in BESW could affect the removal of the two contaminants due to their interactions as well as their simultaneous entry into rhamnolipid solutions affecting the structure of rhamnolipid micelles. The results of FT-IR and NMR indicated varying mechanisms of removing PHE and Cd from co-contaminated soil. Remediation performance of BESW is affected by not only the features of co-existing pollutants but also the experimental conditions. Results of batch experiments showed that pH, temperature, concentration and ionic strength of rhamnolipid solution could have a significant impact on co-contaminated soil remediation of BESW. The Taguchi-based Grey Relational Analysis was employed to determine the optimum conditions for simultaneous removal of PHE and Cd in BESW. The optimization results indicated that the optimum conditions for PHE-Cd-co-contaminated soil remediation using BESW were a pH of 9, a rhamnolipid concentration of 5 g/L, temperature of 15 °C and ionic strength of rhamnolipid of 0.01 M, under which 72.4% of cadmium and 84.8% of phenanthrene were removed.
